# The Neglect and Fast Spread of Some Arboviruses: A Note for Healthcare Providers in Nigeria

**DOI:** 10.3390/diseases6040099

**Published:** 2018-11-05

**Authors:** Olatunji M. Kolawole, Adebimpe A. Seriki, Ahmad A. Irekeola, Jeremiah I. Ogah

**Affiliations:** Infectious Diseases and Environmental Health Research Group, Department of Microbiology, Faculty of Life Sciences, University of Ilorin, Ilorin 240003, Nigeria; pulchritudeadebimpe@gmail.com (A.A.S.); profahmad007@yahoo.com (A.A.I.); ogahij@yahoo.com (J.I.O.)

**Keywords:** arbovirus, epidemiological, mosquito, clinical manifestation, Nigeria

## Abstract

Arboviruses are distributed worldwide and constitute significant health burden globally. Outbreaks of arboviruses have been reported in Africa and beyond. In Nigeria, like in many other countries, arbovirus infections are more often than not neglected. As the early clinical features of arbovirus infections are generally nonspecific, most healthcare providers mistake them for other diseases. Outbreaks have been reported in Africa and beyond. The consequence of missed diagnosis of diseases cannot be overstated. In this review, some epidemiological data, classical syndromes, and risk factors for five human arboviruses (yellow fever YF, dengue DENV, chikungunya CHIKV, Rift Valley fever RVF, and West Nile virus WNV) found in Nigeria are presented. Health practitioners should ensure in-depth analysis rather than a superficial diagnosis of diseases before declaring a course of treatment.

## 1. Introduction

Arthropod-borne viruses are generally referred to as arboviruses. These viruses depend on vertebrate hosts as well as arthropods such as ticks, mosquitoes, insect bugs, sandflies, and midges for their proliferation. Over 500 species of arboviruses belonging to 14 different virus families are known [1]. Although many of these known arboviruses are neither pathogens of humans nor animals [2], over 100 have been associated with human and/or animal diseases [1]. Virtually all the arboviruses that cause human and/or animal diseases belong to one of four virus families: *Bunyaviridae*, *Flaviviridae*, *Togaviridae*, and *Reoviridae*.

Arboviruses range from being regional to global in their distribution. For instance, bluetongue virus, dengue virus (DENV), West Nile virus (WNV) and chikungunya virus (CHIKV) can be found in nearly every part of the world [2]. Meanwhile, in India and Central and Southeast Asia, the mosquito-borne Japanese encephalitis virus is widespread, largely due to circumstances that promote the amplification of the virus in favorable hosts, such as the abundance of *Culex tritaeniorhynchus* mosquito species and pig farming [3,4]. The distribution of arboviruses may be related to the migration and dispersal of their hosts since the biological life cycle (including transmission) of the viruses revolves around the arthropod and vertebrate hosts [5]. While transmission of arboviruses from animals to human (zoonosis) is well-known and documented, transmission from humans to animals (anthroponosis) is quite unfamiliar [6,7]. Similarly, transmission from human to human is uncommon, but could occur. Evidences abound in organ transplantation, blood transfusions, and the use of blood products [8,9,10]. It is therefore pertinent for healthcare practitioners to ensure proper screening of blood and organs before use. The use of contaminated needles from an infected human or animal may also transmit arboviruses [11], thus, putting healthcare workers at risk of infection in areas where arboviruses are widespread among the populace.

In our previous studies we established the presence of arboviruses capable of causing human diseases and belonging to three of the four virus families already highlighted [12,13,14,15,16]. As our findings suggest, DENV is highly prevalent in Nigeria [13]. Despite this revelation, not many healthcare providers, especially those in rural communities, are aware of these arboviruses. In Nigeria, the public health threat posed by arboviruses appears to be greatly underestimated. This is may be connected to the fact that most clinical signs and symptoms of arbovirus infections are non-specific in nature, and could be confused with illnesses like malaria, typhoid, and bacterial meningitis. Our past studies also revealed that diagnosis of arboviral infections are not included in the routine laboratory investigation of ill patients’ samples at various hospitals in Nigeria. The health dangers of arboviruses and their neglect by most healthcare practitioners in Nigeria necessitated this review.

Here, we present some epidemiological data alongside the typical signs and symptoms of five arboviruses: DENV, yellow fever virus (YFV), Rift Valley fever virus (RVFV), CHIKV, and WNV, whose prevalence in Nigeria we have demonstrated in our past studies. These viruses have some similarities and differences with regards to their vector, transmission, nucleic acid, etc. (Table 1). Although most arboviral infections are asymptomatic and often resolve after 1–2 weeks, some arboviral infections could be more severe, resulting in hemorrhage, high fever, encephalitis, meningitis, or even death [2]. The clinical features of some arboviruses found in Nigeria are presented in Table 2. Also, infected arthropods do not show detectable signs of illness. The virus may be in the arthropod for life, and animals and humans infected by arboviruses may suffer mild to severe clinical syndromes [2].

## 2. Yellow Fever Virus Infection

Yellow fever (YF) is a well-known vector-borne disease which poses a considerable health care burden and risk to residents of disease-endemic regions, non-immunized travelers entering disease endemic areas, and persons moving within their own country from low-risk to high-risk regions [17].

Our previous study assessed the prevalence of anti-yellow fever virus (anti-YFV) immunoglobulin G (IgG) antibodies, providing evidence for past exposure to yellow fever virus (either the wild or vaccine strains). It was recorded that regardless of vaccination status of subjects, 21.6% of the subjects enrolled in the study were found to be seropositive to anti-YFV IgG antibodies [12]. This implies that in every five subjects there is approximately one subject who is seropositive for anti-YFV IgG antibodies. The revelation is quite alarming for an infection that has a vaccine. However, our study did not record any positive cases of YFV RNA following RT-qPCR [12]. In a study conducted in Central African Republic, 37% of the participants (including people who reported previous yellow fever vaccination) were confirmed to have YFV antibodies [18]. Sylla et al. (2014) [19] also recorded a prevalence rate of 22.4% anti-YFV IgG antibodies, although this was among non-human primates in southeastern Senegal. Few of the vaccinees had evidence of a yellow card in support of their claim of been vaccinated. There was an overall low anti-YFV IgG seropositivity within the study population, and there seemed to be a delay in the YF outbreak within the study area in several years until after our study was concluded in 2016 [12]. This could be because of herd immunity against yellow fever [17].

Furthermore, it was observed that females were found to be more seropositive for anti-YFV IgG antibodies compared to males. While the reason for this is unclear. Exposure level plays an important role in infection rate. This negates the findings of Niedrig et al. (2008) [20], who reported a slightly higher anti-YFV IgG response in males compared to females who were being evaluated for antibody production following yellow fever vaccination. While studies have shown that a well administered YFV vaccination could confer immunity for up to ten years, various studies have documented the failure of YF vaccination [21,22,23]. There is currently no effective treatment for yellow fever. Immunization is therefore pertinent for preventing and/or lowering morbidity and mortality. The administration of yellow fever vaccine is recommended for persons ≥9 months of age who are living in/or traveling to high risk regions [24]. Despite the availability of vaccines against YFV, there appear to be few people that are positive for anti-YFV IgG antibodies in Nigeria, suggesting a risk for rapid spread of the virus, especially as conditions which promote their distributions are present in Nigeria.

## 3. Dengue Virus Infection

Like YFV infection, people infected with dengue virus are asymptomatic or only present with mild symptoms. However, some individuals enter a critical stage of dengue infection as fever abates [25], with manifestations of the plasma leakage that typically characterizes DHF; skin hemorrhage in the form of petechial, purpuric lesions, and ecchymosis are some common indications. A test for capillary fragility using the tourniquet test could therefore be of help to care-givers. Following fluid leakage from circulation and depletion in blood supply to body organs, shock (dengue shock syndrome) of varying severity as well as a drop in blood pressure may ensue. This critical stage of dengue virus infection is quite rare and mostly occurs in children and young adults [25].

A study by Kolawole et al. (2017 [13]) shows that 46% of the total subjects enrolled in the study were positive for dengue virus IgM antibodies, which is similar to the reports obtained from other studies conducted within Nigeria where prevalence of 51.9%, 30.8%, and 25.7% were reported [26,27,28]. It has been said that variations in climatic conditions and the different geographical locations where studies are conducted alongside extension of urban area into rural area due to the need for food, shelter, and space can affect and influence the rate and prevalence of not just dengue virus but also of other arboviruses like WNV, YFV, CHIKV, RVFV, etc. [29].

In the study, it was noticed that some age groups seemed to be more exposed to the vector of DENV than others. Young adults within the age group 31–40 were the most infected when compared to other age groups, in line with the findings of Idris et al. (2013) [30] where the number of subjects seropositive for dengue type III neutralizing antibodies was the highest in the group aged 30–39. The reason for the high seropositivity in adults could be due to greater exposure of the young adult to the vector in course of searching for means of livelihood. On the contrary, Bello et al. (2016) [26] reported that subjects above 70 years were the most infected with dengue virus. More than half of the subjects who had no form of education were seropositive, corresponding with the findings of Adesina and Adeniji (2016) [28] where individuals with no form of education had dengue infection the most. Aside from exposure to the virus, many factors affect the establishment of the virus in subjects. Some of these factors include body adaptability, level of immunity of the subject, viral load of infection, previous exposure to the virus and members of the genus *Flavivirus*, and the nutritional level of the subjects. 

Self-treatment is very common in Nigeria, especially with the slightest feeling of any feverish conditions, and a study by Idoko et al. (2015) [31] reported seropositivity of DENV in all the subjects under antimalarial medication as compared to those who were not under medication.

## 4. West Nile Virus Infection

The incubation period of West Nile virus (WNV) is about 2 to 15 days, and up to 80% of people infected show no symptoms. In the symptomatic cases, the clinical manifestations could be broadly categorized as neurologic or non-neurologic [32,33,34].

Our previous study conducted in northcentral Nigeria showed serological positivity of WNV in 15 (7.5%) out of 200 subjects enrolled for the study [14]. Also, an earlier study conducted in northeastern Nigeria [35] reported a prevalence of 25% for WNV. In an assessment of mosquito vectors present in our study area, we identified four genera (*Mansonia*, *Aedes*, *Culex*, and *Anopheles*), with *Culex* sp. (54.4%) being the most identified (unpublished data). The high presence of *Culex* sp. could facilitate easy spread of WNV.

## 5. Chikungunya Virus Infection

More than 70% of people infected with CHIKV develop symptoms which typically begin 3–7 days after infection [36]. While headache, digestive illnesses, arthritis, fatigue, and conjunctivitis may occur, acute fever, joint pain, and rash most commonly symptomatize the infection. 

We conducted a seroprevalence study of anti-CHIKV IgG antibody in Ilorin (Kwara state, Nigeria) in some febrile subjects within the state capital. The study showed that 13.0% of the subjects enrolled were seropositive, while 87.0% were seronegative. The low prevalence of the anti CHKV IgG antibody reveals a very low history of chikungunya incidence within this region which could be due to the lack of exposure of patients to the virus. Nevertheless, upon subjecting some of the anti-CHIKV IgG positive samples to PCR screening, approximately 77% were positive for CHIKV RNA. This implies that the virus may actually be in circulation. Furthermore, Baba et al. (2009) [35], reported a higher prevalence of anti-chikungunya antibody (55%) in Maiduguri.

## 6. Rift Valley Fever Virus Infection

The Rift Valley fever virus (RVFV) has an incubation period of 2–6 days. Infected people most commonly do not show symptoms or only present with mild symptoms such as headache, muscle pains and liver abnormalities. Fever, back pain, dizziness, and generalized weakness which resolve within a week of the onset of illness usually occur in people who become ill. In some people, the infection could progress to a more severe form (although rare), with the manifestation of clinical syndrome including maculo-retinitis accompanied by blurred and decreased vision due to macular edema and retinal hemorrhage, encephalitis accompanied by confusion and coma, and hemorrhagic fever accompanied by multiple hemorrhage, thrombocytopenia, hepatitis, and icterus [37]. The rate of fatality resulting from the infection is usually very low in humans.

Olaleye et al. (1996) [38] carried out a study on RVFV in animals of which samples were collected from 6 domestic animals species in Nigeria between 1986 and 1989. The study reported presence of antibodies to RVFV. For about two decades after this report, there appeared to be a long silence in studies on RVFV until the report of Kolawole et al. (2017e) [16] identified an alarming 21.5% prevalence of the virus within Ilorin, Nigeria. At about the same period, outbreak of the virus was reported in Niger State, Nigeria, where 23 deaths were recorded and 60 people hospitalized [39].

## 7. Some Predisposing Risk Factors to Arboviral Infection in Nigeria

➢ **AGE**: Age determines how fast an individual will be susceptible to the viral infections. Children and the elderly are more prone to these infections due to their lowered immunity. Young adults are often more infected because this is the age when an individual starts to search for a means of livelihood; as a result they have constant contact with the vector [14].
➢ **LEVEL OF EDUCATION**: It was discovered that the more educated an individual is the more cautious and protective they are of their immediate environment and health than an individual who is just interested in eating and feeding his immediate family.
➢ **TYPE OF HOUSE**: Those living in crowded houses (e.g., a house with about 12–15 rooms accommodating about 12–15 different families and children, all sharing one or two toilets and a bathroom) with a lack of proper hygiene have a greater risk of been infected than an individual staying in a 2–3 bedroom flat with just his family.
➢ **NUMBER OF PERSONS PER ROOM**: Some houses in developed cities have several rooms with a child or two sharing a room. On the other hand, we have some family of six, seven or even more individuals sharing a room; this increases the rate at which an infection will spread.
➢ **CONTACT WITH LIVESTOCK**: Direct contact with infected blood or organs of animals, especially domestic animals.
➢ **EXPOSURE TO MOSQUITO BITES**: Constant exposure to mosquito bites from the vectors that transmit viral infections could get one infected, especially when the mosquitoes have fed on an infected animal or human blood (Table 3).

## 8. Possible Reasons for Increased Prevalence of Arboviral Infections in Nigeria

**Lack of Awareness about the viruses:** Most health practitioners do not have much information about viruses, and as a result there is streamlined blood screening of febrile patients for the malaria parasite and typhoid, not knowing patients are dying gradually as a result of other viruses.

**Unavailability of Vaccines**: Health care centers and the populace as a whole do not have access to vaccines (e.g., yellow fever virus vaccines) except those that have been released by the ministry in charge of health.

**Priority/Lack of Funds**: Funding is a major challenge in curbing these viruses. Funds are needed for distributing the vaccines and for clearing and making the environment clean so as to inhibit the growth and spread of the vectors. 

**Nonchalant attitudes/Priorities**: Lack of prioritizing has led to the spread and growth of the vectors reaching this level. The moment the governments are ready to wage war against arboviruses, funds will be made available, with policies and frameworks that could help combat these arboviral infections put in place.

**Lack of Access to Diagnostic Tools**: Screening materials for these viruses are not generally available in government-owned hospitals and for individuals. As a result, in rare cases when screening needs to be done, transporting of blood samples is necessary.

**Poverty**: These viruses are more common in rural areas and developing towns and cities where there are conditions favorable for the growth and spread of the vectors, and individuals are unable to afford going to health centers until they are left with no other options. As such, some patients die without having visited a hospital.

## 9. Conclusions 

Given that arboviruses have both regional and global distributions and human infections could be debilitating and fatal, the need for early identification and diagnosis of arboviral infections cannot be overemphasized. Worrisome however, is the neglect of arboviral infections in Nigeria, as well as in some other countries. For instance, based on our findings in 2016, we accentuated the imminence of yellow fever outbreak in Ilorin, Nigeria, owing to the nonchalance of people towards receiving vaccination, low anti-YFV IgG seropositivity, and the speculated circulation of the wild-type YFV [12]. If adequate attention had been given to the early warning, the outbreak of YV experienced in Ilorin, Nigeria, in 2017 [40], may have been averted. Health practitioners should therefore acquaint themselves with the typical clinical signs and symptoms of arbovirus infections in order to avoid missed diagnosis, prevent proliferation of possible diseases, and reduce associated case fatalities.

## 10. Recommendations

The prevention of arbovirus infection is dependent on eliminating the risk factors that promote the survival of vectors responsible for their transmission; it is therefore recommended that stagnant water, bushes, and trees that can serve as their habitat are cleared in order to prevent the prevalence of the vectors.

In areas where there is possibility of an outbreak, residents should be consider these viruses and be encouraged to clear standing water in and around their homes and offices where mosquitoes can breed. Clogged drainages should be cleared, as should any other items like discarded tires that could store up water for the vector breeding.

Hospital laboratories should employ diagnostic tools which could be used in the detection of arboviral infections so as to limit the possibility of missed diagnosis.

Multi-sectorial collaboration and enhanced surveillance are needed for early detection of any arboviral infection in arbovirus-endemic areas.

## Figures and Tables

**Table 1:** Characteristics of some arboviruses.

Virus	Yellow Fever Virus	Dengue Virus	West Nile Virus	Chikungunya Virus	Rift Valley Fever Virus
**Nucleic acid**	Single-stranded positive sense RNA	Single-stranded positive sense RNA	Single-stranded positive sense RNA	Single-stranded positive sense RNA	Single-stranded negative sense RNA
**Number of serotypes**	1	5	1	1	1
**Family**	*Flaviviridae*	*Flaviviridae*	*Flaviviridae*	*Togaviridae*	*Bunyaviridae*
**Principal vector**	*Aedes aegypti*	*Aedes aegypti*	*Culex pipiens*	*Aedes aegypti*	*Aedes vexans* and *Culex tritaeniorhynchus*
**Principal mode of transmission**	Infected mosquito bite	Infected mosquito bite	Infected mosquito bite	Infected mosquito bite	Infected mosquito bite, contact with infected animals, wild fauna, etc.
**Availability of approved vaccine**	Yes	No	No	No	Yes (animal use)

**Table 2:** Clinical features of some arboviruses.

Infectious Agent	Nature of Symptom	Incubation Period	Signs and Symptoms
Yellow fever virus	Mostly asymptomatic	Around 3–6 days	**Initial stage** (Duration: 3–4 days of viremia): - Headache, fever, chills, backpain, fatigue, muscle pain, general body ache, nausea and vomiting. **Toxic stage** (Starts within 48 h after viremia; occurs in approximately 15% of cases): - Periodic fever, jaundice, abdominal pain. - Possibility of bleeding in the eyes, mouth, and gastrointestinal tract. - Permanent damage in organs is rare, and people who survive the infection develop lifelong immunity.
Dengue virus	Asymptomatic or presentation of mild symptoms	Around 4–7 days	**Dengue fever:** - Abrupt high fever (could be over 40 °C (104 °F)) that lasts about 2–7 days. The fever may be biphasic. - Other nonspecific signs and symptoms include headache, nausea and vomiting, weakness, joint pain and body aches. - In up to 50% of people that develop symptoms, rash may occur. - Dengue rash initially appears as flushed skin and later as a measles-like rash. The rash normally starts on the trunk and extends to the face and extremities. - Scattered or confluence petechiae may also appear as temperature subsides to or below normal towards the end of the febrile stage of disease. **Dengue hemorrhagic fever (DHF):** Plasma leakage at a critical stage of dengue characterizes DHF. Manifestations include: - Skin hemorrhage such as petechiae, purpuric lesion, ecchymoses. - Shock (dengue shock syndrome) of varying severity. - Drop in blood pressure may ensue. - The critical stage of dengue is rare and mostly occurs in children and young adults.
West Nile virus	Asymptomatic in up to 80% of infected people	About 2–15 days	**Non-neurologic West Nile virus (WNV) infection** (may be mild or severe, lasting few days to several weeks): - Headache, fever, chills, malaise, anorexia, nausea, vomiting, diarrhea, myalgia, joint pain, retro-orbital pain, and lymphadenopathy. - Rash may develop in a few infected people. - Less often, non-neurologic complications such as pancreatitis, hepatitis, orchitis, myocarditis, hemorrhagic fever, nephritis, and rhabdomyolysis may occur. **Neurologic WNV infection:** - Neurologic WNV infections include three major syndromes—meningitis, encephalitis, and acute flaccid paralysis. - Other signs and symptoms associated with neurologic WNV infection include chills, nausea and vomiting, ataxia, myalgia, arthralgia, rash, tremors, visual disturbance and bulbar dysfunction. - Only less than 1% of infected people develop neurologic syndromes.
Chikungunya virus	Symptomatic in more than 70% of infected people	About 3–7 days	- Acute fever (which may be biphasic, and lasts for about a week), joint pain, and rash, most commonly symptomatize the infection. - Pain around joints such as the shoulders, elbows, ankles, knees, wrists, as well as joints of the hands and feet develop after the fever. - Joint pain may persist for few weeks or even several months or years. - Maculopapular rash may appear two to five days after the manifestation of symptoms. - Other signs and symptoms include headache, digestive illnesses, arthritis, fatigue, and conjunctivitis. - Only less than 1% of infected persons (mainly older adults) die from chikungunya virus infection.
Rift Valley fever virus	Mostly asymptomatic or presentation of mild symptoms	Around 2–6 days	- Headache, muscle pains, and liver abnormalities are mild presentations. Fever, back pain, dizziness, and generalized weakness (resolving within a week of the onset of illness) usually occur in people who become ill. - In some people, the infection could progress to a more severe form (although rare) with the manifestation of clinical syndrome including maculo-retinitis accompanied by blurred and decreased vision due to macular edema and retinal hemorrhage, encephalitis accompanied by confusion and coma, and hemorrhagic fever accompanied by multiple hemorrhage, thrombocytopenia, hepatitis, and icterus. - The rate of fatality resulting from the infection is usually very low in humans.

**Table 3:** Predisposing risk factors to some arboviruses in Nigeria.

Arboviruses	Yellow Fever Virus	Dengue Virus	Chikungunya Virus	West Nile Virus	Rift Valley Fever Virus
Age	+	+	+	+	+
Constant visit to Abattoir	−	−	−	−	+
House type	+	+	+	+	+
Sitting out at nights or day	+	+	+	+	+
Vaccination status	+	+	+	+	+
Mosquito net usage or Insecticides	−	−	−	+	+
Stagnant water	+	+	+	+	+
Bushes around houses	+	+	+	+	+
Dirty environments	+	+	+	+	+
Nature of jobs	+	+	+	+	+

KEY: −: Not applicable; +: Applicable.

## References

[1] Karabatsos N. (1985). International Catalogue of Arthropod-Borne Viruses.

[2] Liang G., Gao X., Gould E.A. (2015). Factors responsible for the emergence of arboviruses; strategies, challenges and limitations for control. Emerg. Microbes Infect..

[3] Takahashi M. (1976). The effects of environmental and physiological conditions of Culex tritaeniorhynchus on the pattern of transmission of Japanese encephalitis virus. J. Med. Entomol..

[4] Campbell G.L., Hills S.L., Fischer M., Jacobson J., Hoke C.H., Hombach J.M., Marfin A.A., Solomon T., Tsai T.F., Tsu V.D. (2011). Estimated global incidence of Japanese encephalitis: A systematic review. Bull. World Health Organ..

[5] Calisher C.H. (1994). Medically important arboviruses of the United States and Canada. Clin. Microbiol. Rev..

[6] Kuno G., Chang G.J. (2005). Biological transmission of arboviruses: New insights into components mechanisms, unique traits and their evolutionary trends. Clin. Microbiol. Rev..

[7] Weaver S.C., Reisen W.K. (2010). Present and future arboviral threats. Antivir. Res..

[8] Iwamoto M., Jemigan D.B., Guasch A., Trepka M.J., Blackmore C.G., Hellinger W.C., Pham S.M., Zaki S., Lanciotti R.S., Lance-Parker S.E. (2003). West Nile Virus in Transplant Recipients Investigation Team. Transmission of West Nile virus from an organ donor to four transplant recipients. N. Engl. J. Med..

[9] Tambyah P.A., Koay E.S.C., Poon M.L.M., Lin R.V.T.P., Ong B.K.S. (2008). Transfusion-Transmitted Dengue Infection Study Group. Dengue hemorrhagic fever transmitted by blood transfusion. N. Engl. J. Med..

[10] Teo D., Ng L.C., Lam S. (2009). Is dengue a threat to the blood supply?. Transfus. Med..

[11] Venter M., Swanepoel R. (2010). West Nile virus lineage 2 as a cause of zoonotic neurological disease in humans and horses in Southern Africa. Vector-Borne Zoonotic Dis..

[12] Kolawole O.M., Irekeola A.A., Seriki A.A., Bello K.E. (2016). Investigation of risk factors associated with malaria and yellow fever co-infection among febrile subjects in Ilorin, Nigeria. J. Med. Soc..

[13] Kolawole O.M., Seriki A.A., Irekeola A.A., Bello K.E., Adeyemi O.O. (2017). Dengue virus and malaria concurrent infection among febrile subjects within Ilorin Metropolis, Nigeria. J. Med. Virol..

[14] Kolawole O.M., Adelaiye G., Ogah I.J. (2017). Emergence and associated risk factors of vector borne West Nile Virus (WNV) infection in Ilorin, Nigeria. J. Arthropod Borne Dis..

[15] Kolawole O.M., Bello K.E., Seriki A.A., Irekeola A.A. (2017). Serological survey of Chikungunya virus in Ilorin metropolis, Nigeria. Braz. J. Infect. Dis..

[16] Kolawole O.M., Ajibola O.A., Ogah I.J. (2017). Serological and Molecular Evidence of Rift Valley Fever in Febrile Malaria Patients 21 years After Last Report in Nigeria. Ann. Sci. Technol..

[17] Gotuzzo E., Yactayo S., Cordova E. (2013). Efficacy and duration of immunity after yellow fever vaccination: Systematic review on the need for a booster every 10 years. Am. J. Trop. Med. Hyg..

[18] Rogers D.J., Wilson A.J., Hay S.I., Graham A.J. (2006). The global distribution of yellow fever and dengue. Adv. Parasitol..

[19] Sylla M., Dubot-Peres A., Sylla E.D.D., Molez J., Ndiaye M., Pourrut X., Gonzalez J. (2014). Yellow fever and dengue fever viruses’ serosurvey in non-human primates of the Kedougou forest galleries in Southeastern Senegal. Afr. J. Microbiol. Res..

[20] Niedrig M., Kursteiner O., Herzog C., Sonnenberg K. (2008). Evaluation of an indirect immunofluorescence assay for detection of immunoglobulin M (IgM) and IgG antibodies against yellow fever virus. Clin. Vaccine Immunol..

[21] Lu S. (2014). Using convalescent whole blood or plasma as passive immune therapy for the global war against Ebola. Emerg. Microbes Infect..

[22] Tuboi S.H., Costa Z.G., da Costa Vasconcelos P.F., Hatch D. (2007). Clinical and epidemiological characteristics of yellow fever in Brazil: Analysis of reported cases 1998–2002. Trans. R. Soc. Trop. Med. Hyg..

[23] Reinhardt B., Jaspert R., Niedrig M., Kostner C., L’age-Stehr J. (1998). Development of viremia and humoral and cellular parameters of immune activation after vaccination with yellow fever virus strain 17D: A model of human flavivirus infection. J. Med. Virol..

[24] Staples J.E., Gershman M., Fischer M. (2010). Yellow fever vaccine: Recommendations of the Advisory Committee on Immunization Practices (ACIP), Centers for Disease Control and Prevention—Morbidity and Mortality Weekly Report (MMWR). Recomm. Rep..

[25] Gubler D.J. (1999). Dengue and dengue hemorrhagic fever. Clin. Microbiol. Rev..

[26] Bello O.A., Aminu M., Jatau E.D. (2016). Seroprevalence of IgM Antibodies to Dengue Fever Virus among Patients Presenting with Symptoms of Fever in Some Hospitals in Kaduna State, Nigeria. Int. J. Sci. Res..

[27] Faneye A., Idika N., Motayo B.O., Adesanmi A., Afocha E. (2013). Serological Evidence of Recent Dengue Virus Infection Among Febrile Children in a Semi-Arid Zone. Am. J. Infect. Dis..

[28] Adesina O.A., Adeniji J.A. (2016). Incidence of Dengue Virus in febrile episodes in Ile-Ife, Nigeria. Afr. J. Infect. Dis..

[29] Baba M.M., Marie-Francois S., Vorndam A.V., Adeniji J.A., Diop O., Olaleye D. (2009). Dengue Virus Infections in Patients Suspected of Malaria/Typhoid in Nigeria. J. Am. Sci..

[30] Idris A.N., Baba M.M., Thairu Y., Bamidele O. (2013). Sero-prevalence of Dengue type-3 virus among patients with febrile illnesses attending a tertiary hospital in Maiduguri, Nigeria. Int. J. Med. Sci..

[31] Idoko O.M., Ado S.A., Umoh V.J. (2015). Prevalence of Dengue Virus and Malaria in Patients with Febrile Complaints in Kaduna Metropolis, Nigeria. Br. Microbiol. Res. J..

[32] Gould E.A., Solomon T. (2008). Pathogenic Flaviviruses. Lancet.

[33] Kramer L.D., Li J., Shi P.Y. (2007). West Nile virus. Lancet Neurol..

[34] Petersen L.R., Brault A.C., Nasci R.S. (2013). West Nile virus: Review of the Literature. JAMA.

[35] Baba M., Logue C.H., Oderinde B., Abdulmaleek H., Williams J., Lewis J., Laws T.R., Hewson R., Marcello A., Agaro P.D. (2013). Evidence of arbovirus co-infection in suspected febrile malaria and typhoid patients in Nigeria. J. Infect. Dev. Ctries..

[36] Thiberville S., Moyen N., Dupuis-Maguiraga L., Nougairede A., Gould E.A., Roques P., Lamballerie X. (2013). Chikungunya fever: Epidemiology, clinical syndrome, pathogenesis and therapy. Antivir. Res..

[37] Chevalier V., Pepin M., Plee L., Lancelot R. (2010). Rift Valley fever—A threat for Europe?. Euro Surveill..

[38] Olaleye O.D., Tomori O., Ladipo M.A., Schmitz H. (1996). Rift Valley fever in Nigeria: Infections in humans. Rev. Sci. Tech..

[39] International Federation of Red Cross and Red Crescent Societies Niger Red Cross Responds to Rift Valley Fever Outbreak. https://reliefweb.int/report/niger/niger-red-cross-responds-rift-valley-fever-outbreak.

[40] Nigeria Centre for Disease Control (NCDC) (2017). Strengthening surveillance for yellow fever. Wkly. Epidemiol. Rep..

